# The use of digital twins in healthcare: socio-ethical benefits and socio-ethical risks

**DOI:** 10.1186/s40504-021-00113-x

**Published:** 2021-07-05

**Authors:** Eugen Octav Popa, Mireille van Hilten, Elsje Oosterkamp, Marc-Jeroen Bogaardt

**Affiliations:** 1 Department of Communication, Philosophy and Technology, Wageningen University, Wageningen, the Netherlands; 2 Wageningen Economic Research, The Hague, the Netherlands

## Abstract

Anticipating the ethical impact of emerging technologies is an essential part of responsible innovation. One such emergent technology is the *digital twin* which we define here as a living replica of a physical system (human or non-human). A digital twin combines various emerging technologies such as AI, Internet of Things, big data and robotics, each component bringing its own socio-ethical issues to the resulting artefacts. The question thus arises which of these socio-ethical themes surface in the process and how they are perceived by stakeholders in the field. In this report we present the results of a qualitative study into the socio-ethical benefits and socio-ethical risks of using digital twins in healthcare. Employing insights from ethics of technology and the Quadruple Helix theory of innovation, we conducted desk research of white literature and 23 interviews with representatives from the four helixes: industry, research, policy and civil society. The ethical scan revealed several important areas where the digital twin can produce socio-ethical value (e.g., prevention and treatment of disease, cost reduction, patient autonomy and freedom, equal treatment) but also several important areas of socio-ethical risks (e.g., privacy and property of data, disruption of existing societal structures, inequality and injustice). We conclude with a reflection on the employed analytical tool and suggestions for further research.

## The digital twin finally peaks

The idea of replicating humans is anything but new. Descartes was perhaps the first to ponder philosophically on the idea of such “automata” (Descartes 1980: 32) and contemporary philosophy is in fact replete with twin-like creatures such as “doppelgangers” (Putnam 1996), “zombies” (Kirk 2008), and “swamp-men” (Donald 1987). Interesting and controversial as such thought experiments might be, they are nothing but philosophical devices – they are sometimes called “intuition pumps” because they are devised to trigger and direct our intuition (Dennett 2013). But nowadays, such improbable twin versions of humans are approaching reality. Recent research on the topic of ‘digital twins’ has created the possibility of a dynamic (i.e., constantly updated) replica of the human body or at least some parts of the human body (Bagaria et al. 2020; El Saddik 2018; Jimenes et al. 2020; van Houten 2018a). A digital twin is not a robot, but the basic idea still is that of Descartes: it is a system that replicates in some way an individual, a part of an individual or a set of individuals. Various definitions of digital twins have been proposed nowadays (see overview in Fuller et al. 2020), but there is a core idea captured by most of them.

A digital twin is [ … ] a living model of the physical asset or system, which continually adapts to operational changes based on the collected online data and information, and can forecast the future of the corresponding physical counterpart (Liu et al. 2018)

Historically, the expression ‘digital twin’ is related to the nowadays more common notion of a ‘digital model’.^1^ As early as 1960, radiologists have started using rudimentary computational models of phantoms. Phantoms are objects employed in medicine or other fields to replicate the reaction of human tissue to certain processes, e.g., to radiation. The digitalization of phantoms resulted in *digital phantoms*. The digital phantom is in many ways the ancestor of the digital twin. It is then not surprising that the earliest use of the expression *digital twin* appeared in 1994 in the field of medical imaging (Renaudin et al. 1994). The term ‘digital twin’ was later used in other fields such as aerospace engineering where it stood for an imagined “ultrarealistic in geometric detail, including manufacturing anomalies, and in material detail, including the statistical microstructure level” (Tuegel et al. 2011: 2). It is common for studies of digital twins nowadays to trace the ancestry of the term to this more recent use in engineering (Chen 2017; El Saddik 2018; Vathoopan et al. 2018).

As the definition above suggests, a digital twin is more than just a digital *model*. This difference is captured by the word ‘living’ in the definition above which implies that a digital twin is connected to the real-life counterpart in a way a mere model is not (Kritzinger et al. 2018). The continuous adaptation of the twin to the real-life counterpart is helped by various technologies such as sensors, high-speed communication, cloud computing, artificial intelligence and many more (Raden 2020). The digital twin is therefore not one technology but a *technological cocktail*. Digital twins have been recently been named among the “ten most strategic emerging concepts for the coming years” and expectations have been formulated that the technology will lead to spending of $10.96 billion in 2022 (Saracco 2018).

In healthcare, the following fields are expected to be impacted by the advent of digital twins: personalized and precision medicine (Harris 2020), “to build biologically detailed digital reconstructions” of a brain or a heart (van Houten 2018b), particular models for specific conditions such as brain aneurisms (Shugalo 2019), simulation models for operations and other interventions by using the ‘-omics’: “genomics, biomics, proteomics, or metabolomics, as well as physical markers, demographic, and lifestyle data over time of an individual” (Raden 2020), drug discovery through in silico (‘organ-on-a-chip’) clinical trials (Shugalo 2019).

In this paper, we focus on the use of digital twins in healthcare, an area that has already received some recent attention from ethicists and other scholars (Bagaria et al. 2020; Björnsson et al. 2020; Bruynseels et al. 2018). The use of digital twins in healthcare has prompted not only significant hopes for the improvement of diagnostics and treatment, it has also sparked debates about its social and ethical consequences (e.g., Bruynseels et al. 2018; Bruynseels 2020). To start with, digital twins inherit most of the socio-ethical issues concerning privacy and individuality (our conception of a human individual) that have been associated in the past with personalized medicine and health (Korthals 2008). It is unclear whether the digital twin will eventually exacerbate or alleviate such already-existing concerns. As a technological cocktail, however, it is fair to presume that a digital twin will trigger more, rather than fewer, areas of dialogue. The time is therefore ripe for a socio-ethical scan of the use of digital twins in healthcare.

## Socio-ethical scan for the emerging digital twin

Ethical assessment of technology is nowadays central to a variety of disciplines such as responsible research and innovation (Owen et al. 2013; van den Hoven 2013), technology assessment in its various versions (Cotton 2014; Grunwald 2009; Guston and Sarewitz 2002), and value-sensitive design (Friedman and Hendry 2019; van den Hoven 2013). The goal of such an assessment is to detect and eventually prevent a technology’s negative impact on society but also to allow stakeholders from various sectors of society to reflect and deliberate on the resulting cost-benefit calculations. This goal is in line with a larger cultural vision of science and technology as democratized territories where systemic change, as that incurred by technology, is first subjected to a critical scrutiny and dialogue in light of its predicted socio-ethical impact (von Hippel 2005; Stirling 2005).

Such ethical assessment of technology is of particular interest in the case of *emerging* technologies (Brey 2012; Lin 2010; Lucivero et al. 2011; Marchant et al. 2011; Sandler 2014; Swierstra and Rip 2007). Emerging technologies are assumed to be more malleable in the early stages of their development given that the chain of decisions that leads from fundamental research to concrete artefacts has yet to fully unfold (Collingridge 1982). A second reason for the particular interest in the ethical assessment for emerging technologies is the prospect of curbing the technology’s trajectory before it impacts society. Emerging technologies can be modified more easily because the design has yet to be fully established and the manner in which they impact society can, at this stage, be more easily controlled. When dealing with emerging technologies, some approaches take a more explorative, grasp-in-the-dark type of investigation of socio-ethical impact. A traditional example is the *ethical matrix* (Mepham 2010). The ethical matrix and related tools such as the ethical grid and the ethical Delphi are fairly general instruments that have been developed for usage in virtually all areas of R&D (see Cotton 2014)*.* Other approaches propose checklists of ethical issues for *specific* technologies (e.g., Hofmann 2005). For the topic of digital twins, a method is needed that is neither too general nor too specific, in order to both be sensitive to more general trends and to capture the specificity of digital twins (and in this case even more specifically their use in healthcare).

The *anticipatory technology ethics* approach (ATE) seeks to integrate the dimensions of both ethical assessment and foresight from past approaches (Brey 2012, 2017; Stahl et al. 2016). The approach proposes a series of categories that can be employed both to assess the current status of a particular technology and to engage in various forms of forecasting. Brey proposes that the categories to be used in anticipatory technology assessment are the following broad categories: ‘Harms and Risks’, ‘Justice’, ‘Rights’ and ‘Well-being and Common Good’ (Brey 2012: 12)*.* Each of these four categories is further split into several items, such that, for example, the ‘Harms and risks’ main category comes to include the following sub-categories ‘Health and bodily harm’, ‘Pain and suffering’, ‘Psychological harm’, ‘Harm to human capabilities’, ‘Environmental harm’, ‘Harms to society’*.* These categories establish for our purposes an optimal level of generality. They are not too close to the empirical reality (with the risk of becoming technology-specific), nor are they too far from this reality (with the risk of becoming abstract and all-embracing).

Methodologically, however, a problem we noticed with these categories is that they are not mutually exclusive. While we can accept Brey’s remark that their partial coverage is somewhat inevitable (since new technologies will always create new issues), we see the need to intervene in this list with regard to its internal consistency. As a heuristic tool, the category ‘Harm and Risk’ is, to start with, broad enough to cover most other categories. An undesirable limitation on freedom, which would fall under the Brey category of ‘Rights’, can in fact easily be described as a harm and even as a risk. Further, looking into the respective sub-categories, we notice that ‘Pain and suffering’ overlaps with other types of harm, such as ‘Psychological harm’. A problem coming from the opposite direction, so to speak, is that defining categories into sub-categories and sub-sub-categories can function as a limiting definition of a category whereas respondents might benefit from the freedom to discover unexplored semantic areas of a certain sub-category. For example, we find under the category of ‘Rights’ and the sub-category of ‘Freedom’ the following sub-categories: (1) Freedom of movement; (2) Freedom of speech and expression; (3) Freedom of assembly. Of course, these are important categories, but if the suggestion is given that these three fully capture the concept of ‘freedom’, then the tool loses its applicability in a context where ‘freedom’ might mean, e.g., “Freedom to choose healthcare” or “Freedom to decide when to make a doctor’s appointment”.

We therefore suggest that the ‘Brey categories’ be rendered in a form that minimizes their internal overlap and thus increases their consistency. This is particularly necessary in qualitative research, where interviewees are more likely to open up meta-discussions about the tools employed, e.g., “Are A and B the same thing? What is the difference between them? Etc.” We would therefore like to avoid such overlaps as much as possible without unduly limiting the range of the toolkit as a whole. In Table 1 below we present the modified version of the Brey categories that we employed for the present research. While not all possible overlap was solved, the elimination of some categories and re-formulation of others lead to a more systematic tool that offers fewer spaces for meta-discussions.

**Table 1:** The Brey categories abridged

• Impact on individuals ○ Health and body ○ Pain and suffering ○ Psychological effect ○ Effect on human capabilities ○ Environmental impact	• Justice ○ Just distribution of primary goods, capabilities, risks and hazards ○ Nondiscrimination and equal treatment relative to age, gender, sexual orientation etc. ○ North–south justice ○ Intergenerational justice
• Rights ○ Freedom ○ Autonomy ○ Human dignity ○ Privacy ○ Property ○ Other basic human rights as specified in human rights declarations (e.g., to life, to have a fair trial etc.) ○ Animal rights and animal welfare	• Well-being and the common good ○ Happiness and virtue ○ Vital social institutions and structures ○ Democracy and democratic institutions ○ Culture and cultural diversity

## Method

Our research question was: What are the most prominent socio-ethical benefits and socio-ethical risks triggered by the advent of the digital twin in healthcare and how do stakeholders perceive these benefits and risks? The question lends itself to *qualitative* study since we do not seek to trace the frequency or weight of values and risks but rather to understand how stakeholders perceive and formulate them in the context of the selected emerging technology.

In order to answer the research question, we first carried out preliminary desk research on gray literature (industry and governmental reports), academic literature (Scopus), and journalistic outputs (news reports, blogs posts, industry news). This preliminary research was intended to familiarize ourselves with already existing claims regarding values and risks but also to investigate the extent to which these values and risks are brought to light and reflected upon.

Following the preliminary desk research, a series of semi-structured interviews were carried out with representatives of the four major societal sectors: (i) industry and business, (ii) civil society, (iii) policy and (iv) research. We draw these notions from the quadruple helix theory of innovation, each of the four sectors representing one helix (Carayannis and Campbell 2009; McAdam et al. 2018; Monteiro and Carayannis 2017). For the selection of candidates, we took the more recent *processual* understanding of the quadruple helix theory, meaning that the four categories are not interpreted as groups but rather as four types of value creation processes (García-Terán and Skoglund 2019; McAdam et al. 2018). Each process is different from the other three not because the individuals have different titles or educational backgrounds but because their activity is rewarded differently. Industry is directed towards business value, civil society towards social value, policy towards political value and research towards academic value. Philosophically, the idea can be traced back to Walzer’s *Spheres of Justice,* according to which what counts as ‘good’ (or beneficial, just, valuable etc.) does not have universal traits but rather must be distinguished according to specific social spheres that communicate with each other but cannot be reduced to one another (Miller and Walzer 1995; Walzer 1983).

This processual interpretation of the helixes influences the selection of candidates for the interview because a person’s day-to-day activities become more relevant for her placement in a certain category than the person’s title or background. For our purposes, we aimed to obtain a relatively equal distributions of representatives per helix in order to ensure the diversity of the discussions and avoid saturation. However, this was not a sine qua non condition given that no quantitative claims were aimed at in this study. In Table 2, we indicate the type of institution, gender and helix for each respondent.

**Table 2:** Respondents and their respective helix

Respondent	Type of institution	Gender	Helix
1	Tech	M	Industry
2	Tech	M	Industry
3	Software	M	Industry
4	ICT & Consulting	M	Industry
5	Software & Consulting	F	Industry
6	Software & Research	F	Industry
7	Ministry	M	National Policy
8	Region	F	Regional Policy
9	University Hospital	M	Research
10	University	M	Research
11	Research Institute	M	Research
12	University Hospital	M	Research
13	University	M	Research
14	University	M	Research
15	Research Institute	M	Research
16	Research Institute	M	Research
17	Research Institute	M	Research
18	Citizen	M	Civil Society
19	Citizen	F	Patient
20	Citizen	F	Patient
21	Citizen	M	Civil Society
22	Public Engagement Organization	F	Civil Society

Each interview lasted between 40 and 70 min. The interview guide consisted of the following parts: (i) introduction in which participants introduce themselves, their organization and their relationship to the digital twin (10 min); (ii) global view of the digital twin concerning future directions, hopes and fears; (iii) socio-ethical scan in which parties were asked to select items from the (abridged) Brey categories in order to identify both benefits and risks; (iv) discussion of how to minimize socio-ethical benefits while maximizing socio-ethical risks. In preparatory documents, we also sent the following definition of the digital twin to the interviewees, adapted after a 2018 definition of the European Research Consortium on Information and Mathematics: “A digital twin is a digital replica of real world devices, processes or even persons. The technology draws on domains like machine learning, artificial intelligence and software analytics to provide a dynamic digital representation of its physical counterpart. Thereby, it uses data provided for example by Internet of Things (IoT) sensors as well as information coming from past machine usage and human domain experts”. The use of this definition was an important tool for setting the boundaries of the interview on an emerging technology. Since the technology is emerging, we expected the semantics of the term ‘digital twin’ itself is emerging in the sense that different people might apply the term differently. By employing the same definition for each interview, we ensured that the group was, at least as a matter of starting point, sharing this meaning of the expression.

At the end of each interview we offered an ‘open space’ where the participants could add something that crossed their mind during the interview, a topic that we did not cover and other miscellaneous topics.

All interviews took place online and were visually recorded, with the exception of one where the recording could not be retrieved afterwards for technical reasons. For this specific interview, we relied on notes. The recordings were transcribed *ad litteram* and were coded according to the Brey categories but also using open coding for new themes or areas of socio-ethical discussion. Four researchers coded interviews separately, each focusing mainly on the interviews they have carried out themselves. The resulting analysis was then discussed by all researchers involved and checked for completeness and accuracy. The results of the interviews were then discussed with a series of experts during a 1,5 h workshop with 20 participants. The workshop was focused on the following question: “What can be done in order to increase benefits and decrease risks when using digital twins in healthcare?” The discussion was also recorded and coded for the policy proposals advanced during the discussion. The results from this discussion are processed into the last section of the following chapter.

## Results

In this section, we present the results from the interviews and the workshop. The ‘preliminary topics’ are two topics that arose from the interviews and were picked up by open coding: the meaning of the expression ‘digital twin’ and the projected future of the technology. Following this, we tackle the ‘benefits’ and ‘risks’ that are the main topic of the present study. Results section ends with a series of policy claims regarding the governance framework in which benefits can be maximized and risks can be minimized in the further implementation of digital twins in healthcare.

### Preliminary topics

#### The use of the term ‘digital twin’

It is clear that the term ‘digital twin’ is picking up fast. Industry reports announce the advent of digital twins (Fuller et al. 2020; Kritzinger et al. 2018; Liu et al. 2018), market reports place the technology “among the ten most strategic concepts for the coming years” (Saracco 2018) and, as one of our respondent remarked, the term appears nowadays “in just about every research proposal” connected to digitalization in health. At the same time, respondents have confessed it difficult to determine the scope of such claims given that the meaning of the term ‘digital twin’ can vary widely. Thus, although respondents have generally accepted the definition we have given them as a starting point (see the same one used in The Digital Twin Finally Peaks section), the term’s furry edges become apparent later on during the discussion. Several variants of these discussions are worth noting.

To start with, there is a certain sense in which a digital twin is perceived as being *impossible.* There is, after all, a theoretical limit to the degree of precision you can achieve by representing objects. The term ‘digital twin’, while not yet a misnomer, should not to be taken at face value suggesting a near-exhaustive correspondence between the individual and the copy. The quotes below illustrate this struggle with what is not only a practical limitation but a logical one.

You cannot measure everything on a person. To some extent these digital twins will be confined to a number of parameters.

Yet in the opposite direction, a more lax definition of the term ‘digital twin’ allows for less-than-perfect, less-than-complete and less-than-dynamic copies of the real-life counterpart. According to this interpretation, an *actual* digital twin constantly updated in real time is ‘an overkill’ and perhaps an unnecessary one:

I think that in much healthcare, the pure definition is an overkill. In very many uses such as in silico clinical studies you don’t need the [real-time] link with the patient. In preparing operations and seeking alternative treatment strategies, in all those applications you don’t need a direct link.

The metaphor of a ‘twin’ allows in any case such loose applications and some respondents mentioned that the term often appears as an umbrella term for every effort to digitalize the human body, i.e., to use computer models and simulations in health. In fact, one of our respondents pushed the term to its limits: The use of GPS applications such as Google Maps creates a digital version of several aspects of your behavior that is not fundamentally different than any other (equally imperfect) representations dubbed digital twins. For obvious reasons, as our respondent noted, no one would use the term ‘digital twin’ in this way, but the point remains that a loose interpretation opens the door for any number of applications to deserve the label. This loose interpretation also has its ‘enemies’ who seek to combat such a wide stretch of the meaning. As one industry report put it: “A digital twin is *not* an in silico friend to entertain you or keep track of your tasks. A digital twin is an engineering concept, where individual physical devices are paired with digital models that dynamically reflect the operation and state of those devices (Raden 2020). In many of our interviews the discussions swayed naturally towards these applications that, if not yet digital twins, announce it in a characteristic way. Discussions of various mobile applications and ‘wearables’ as well as, more specifically to health, sensors for various physiological data, are examples of such digital proto-twins. In one industry case, the term ‘digital twin’ was widely advertised by a company that clearly does not need (or want) to go in the direction of a comprehensive view of patients but rather focus on a small number of variables that matter for specific type of diagnosis.

To complicate matters even further, the term ‘digital twin’ is sometimes used to stand for a certain method or approach rather than a specific artefact. Particularly in the manufacturing industry, the term ‘digital twin’ seems to stand for a certain approach to manufacturing and testing rather than discretely for certain types of high-quality, dynamic representations (Tao et al. 2018). In this interpretation, the term appears as a designator of an approach or a methodology rather than the name of an artefact (Stojanovic and Milenovic 2018). In line with this, some of our respondents declared that they do not make any distinction between ‘digital twins’ and other related technologies that are labelled differently but follow the same approach – for example, one of our respondents mentioned ‘digital avatars’ and ‘virtual humans’ as being indistinguishable from ‘digital twins’ at least in the assumed approach to digitalization in health. Similarly, various uses of bio-informatics for in silico treatment will by default count as part of the digital twin approach (or paradigm) without necessarily resulting in the construction of a digital twin as such. A consequence of all this is that it is difficult to assess the status of this digital transformation. For example, one of the respondents who was engaged in evaluating research proposals in the area of health confessed that the term appears in ‘just about every proposal I see’ and seems applicable to a wide variety of topics from “strengthening dikes to elevated sea level, food, and agriculture” but that it is difficult to say what all these proposals have in common.

#### Predictions for the future

In healthcare, the digital twin triggers and will continue to trigger a *linear, evolutionary* change, meaning that the technology comes to accelerate already-existing trends rather than produce a revolutionary switch of direction. This is partly due to the nature of the digital twin as technological cocktail, bringing together already existing technologies (see introduction), but also partly due to the fact that the digitalization process is now anything but new.

I don’t think the [digital twin] is a revolution, I think it is an *evolution.* I don’t know if it is a game changer – I have my doubts about that. There is so much going on already.

Nonetheless, this slow-but-steady change is extraordinarily complex. Although this might be an inherited effect of the broadness of the label ‘digital twin’ itself, respondents generally found it difficult to capture the future of the digital twin. We might infer that our broad cross-sectoral take on the matter was somewhat surprising to our respondents who do not see themselves – at least not in their day-to-day business - as participating in a common transition towards the digital twin. The future of the digital twins hangs in the air in several domains.

First, there is the generalization of already existing application. If some decades ago the computer simulation of the human body was limited to some organs or some processes, the future will bring down barriers on what can be replicated and simulated. Thus, the digital twin industry is rapidly moving from being a niche effort focused, e.g., on a certain organ or physiological process, to being the standard approach to diagnosis and treatment. Second, there is the improvement in the quality of the digital twin. Whether the social and economic costs necessary for this improvement are worth the benefits is sometimes called into question, but there was a consensus on the idea that a digital twin will keep getting better as a diagnosis and treatment tool. That said, there was noticeable difference among respondents concerning the areas that will bring this improvement in real life. Those engaged in modeling tend to focus on the better models that will come while those engaged in data-gathering devices (sensors) tend to focus on the better devices. The co-dependence of ‘good data and good modelling’ was nevertheless implicitly or explicitly acknowledged. How will this improved digital twin look like? On the mathematical side of the modelling, there is little controversy: the digital twin will be a highly complex software that will eventually involve machine learning or other forms of artificial intelligence. On the practical side of data gathering, there is some discussion. Some see the digital twin of the future as a ‘mild’ form of a chip under the skin or an ingestible sensor, both of which are projected to become increasingly small and thus uninvasive. Others have a more holistic vision of the digital twin as a full picture of a person - the fuller the picture the better the twin – such that data is constantly and seamlessly flowing towards the digital counterpart. Once this seamless flow of data is achieved, two respondents noted, the digital twin will act as an accelerator for the contemporary switch of focus from treatment to prevention, meaning that the main function of the data will be to *avoid* contact with the physician rather than improve this contact.

The future will bring a digital consultation process, but when it is really necessary, then doctors will pay a lot of attention to you. Because nobody wants to end up in the doctor’s office. [ … ] The doctor also wants to direct her attention to the right issues and people who really need her.

The improved digital twin will thus not only improve treatment but also work as an improved ‘filtering mechanism’ that would contribute to a lowered burden of disease. As for the area where digital twins will advance the fastest, respondents presented conflicting visions. For some, some fields such as cardiology have the ‘advantage’ of creating a much pronounced need for data and real-time optimization. The field of oncology, however, has the counter advantage of being able to gather data much more easily since patients who have received a cancer diagnosis are less inclined to think about privacy or comfort. (One participant formulated this starkly by saying “data protection is something for healthy people”). Continuing the comparison, organ-level replicas and implants have the advantage that the insights they produce as well as their applications are potentially very broad – not necessarily limited to specific diseases or treatments. The existence of these competing advantages makes it very difficult to predict where the digital twin will ‘strike’ the hardest in the upcoming decades.

We must also mention that not all stakeholders saw the advent of the digital twin as something inevitable. Three of our respondents were skeptical about the capabilities of the digital twin two make a difference in any foreseeable future. Asked whether digital twins will be widely used in the future, one of them expressed himself in no ambiguous terms:

The answer is no. Because of the health system, which is very traditional. And because of the medical doctors who are also highly traditional. So I see a future of let’s say, a niche for digital twins, for a very small group of diseases and patients and medical professionals and hospitals, so at least at the beginning.

For comparison purposes, consider the following more optimistic vision of the future of the digital twin in which each individual becomes a particular data set:

I know for certain that we are going in the direction of this digital twin. The patient will be able to say: “OK, this is my data set gathered from my entire life. What’s happening? What’s changed? What can you do?”

### Socio-ethical value

#### Better prevention treatment and understanding of disease

Without exception, all our respondents mentioned the social benefit of improving healthcare processes. The digital twin is seen as having a positive impact on individual happiness through improved prevention, diagnosis, drug discovery or treatment. The digital twin is perceived as capable of producing such social value by replacing healthcare processes that are not tailored to the individual or because they are carried out intuitively. As one industry report put it, the digital twin promises to replace “subjective data” with “objective data” (Harris 2020). The social benefit of the digital twin thus comes with a critique of already existing healthcare processes. The following quote illustrates this critique in the case of drug delivery:

What we see is that the efficiency of medication is very different within groups – some have very positive results, others have negative ones. And that has to do with the fact that pills are made in batches of millions or in any case thousands. But if you measure individually what everybody needs, it doesn’t make sense to use the 500mg or the 1000mg pill. You notice all of a sudden that person A needs 681mg while the remaining 319mg is just creating more adverse effects.

The same critique is presented by stakeholders in the case of other healthcare processes such as diagnosis. The general idea of establishing the health status of a patient by measuring blood pressure and asking qualitative questions is seen as outdated. The time appears to be ripe for a replacement of these traditions with a more precise, quantitative evaluation of human health and sickness. Some stakeholders refrain from making such general claims and keep it specific. For example, the AI component of a digital twin can help the practitioner in the specific task of detecting tumors in medical images, but it is not always seen as expedient that the technology eventually replaces the ‘human’ component in such visual evaluations.

For some little-understood diseases such as cancer or auto-immune disease, there is a more fundamental need to *understand* the diseas*e* first - to know the enemy before any strategy is being undertaken. Four of our respondents were involved, coming from different helixes, in cancer research, and all of them stressed that the digital twin can produce this more fundamental benefit of *understanding* cancer with all the involved benefits for individual patients.

The level of knowledge of cancer biology was indeed increased in the last ten to twenty years, but is still very low. Many types of cancer are not so well known or understood from a biologic perspective. I think we cannot achieve better results if we are not be able to have a better understanding of the biology of the cancer itself.

#### Cost reduction

Another important benefit is that of cost reduction. Cost reduction can come from a variety of sources, the following categories being the ones most prominently mentioned. First, within certain healthcare institutions, costs can be avoided by performing timely maintenance on equipment and ensuring that the equipment follows the workflow within a medical facility and thus saving costs through better time management (van Houten 2018a). Second, costs can also be reduced by reducing the time-to-market and cost-to-market values of specific drugs and thereby increasing investment opportunities and driving the price of medicine production down. Third, costs can be reduced by shorter treatment periods and error avoidance in both diagnosis and treatment. Fourth, costs can be reduced through patient typing and by dealing with some of the less urgent cases outside the physician’s office – for example by allowing patients to self-diagnose or self-treat. Fifth, the digital twin can also contribute to cost reduction by speeding up processes that in vivo might require more human intervention and more raw materials.

The possibility that these saved costs appear elsewhere in the supply chain is also mentioned yet we have heard conflicting views on whether the digital twin will eventually reduce or increase the general burden of disease (the total impact of a health problem). After all, model development and maintenance are highly technical areas in which cost reduction cannot be easily achieved and, typically, state-of-the-art scanning equipment is particularly costly. On the other hand, medical technologies can be cost-reducing, so eventually it might be very case-specific whether a technology is cost-reducing or cost-increasing. In calculating the costs, one of our interviewers noted, one should not forget that the digital twin, and the digitalization process more generally, comes with an energy price as most computing solutions require high levels of data transfer and storage.

#### Patient autonomy and freedom

The use of digital twins can also allow both patients and healthy individuals to exercise a greater degree of autonomy over healthcare decisions - whether and when to go to the doctor, how to take treatment, what to share with the physician and other stakeholders, and how to respond to diagnostic and health advice. In fact, once more data is available and visible to both the patient and the professional, the relationship between the two changes for not only the professional but also the patient can make more informed decisions regarding courses of action. But the use of digital twins is primarily expected to increase autonomy in the case of prevention, for decisions that used to be in the hands of medical experts can now be ‘pushed back’ to those patients that have the necessary data. For example, if healthy lifestyle was a matter of expert evaluation (e.g., by looking at blood sugar level or blood pressure), the advent of wearables and other sensors has brought about the situation that patients get a better grip of their own lifestyle – or, at least, the data generated by their lifestyle.

If you suffer from a chronical condition, you might not need to go to the hospital but you might be able to apply the treatment at home. That increases your freedom as well as your autonomy over your own life. You are free to choose when you receive the treatment because you’re not dependent on that appointment at the hospital anymore.

Patient autonomy is also linked to the aforementioned advances in disease diagnosis and patient treatment. In some cases, interviewees mention autonomy (and sometimes freedom) as a result of being healthy, the latter being the contribution of the digital twin. However, this is not a result from the specific qualities or technical features of the digital twin but rather from the more general positive outcome of improved healthcare. By contrast, more clearly related to the specificity of the digital twin is the ability of the technology to bring data regarding the patient *closer to the patient* by allowing patients in this way to own and control their biological data. The current distance between the patient and their personal data (to which they have access only after passing through the system’s gatekeepers), is then diminished if the same data can be seen, updated in real time, through an app or some similar cloud application.

#### Other forms of socio-ethical benefits

Other socio-ethical values have been mentioned during the interviews. These have generally been given less attention, either by not being mentioned first or by describing them as adjacent advantages rather than goals in themselves. The most notable are:

- the equal treatment of patients relative to race, gender etc. (through a digitalization of the consultation process or through tackling the bias towards white, healthy, male patients in clinical trials and eventually through non-discriminatory treatment)
- less animal suffering (due to organ-on-a-chip and other in-silico applications related to the digital twin)
- less pain and suffering through less invasive consultation processes
- the possibility of citizens to contribute to science by sharing (or selling) data

### Socio-ethical risks

#### Content, ownership and quality of data

The topic of privacy has been mentioned one way or another in *all* our discussions with stakeholders. The violation of privacy seems to be the most important socio-ethical risk. This corelates with claims from the academic literature where scholars note the negative implications “of a healthcare organization, insurance company, or any other organization having a persistent, detailed picture of biological, genetic, physical, and lifestyle information of a person over time” (Raden 2020). For some of our interviewees, privacy was the main reason why the digital twins might be disadvantageous, all things considered; for others, it was an overstated risk with little impact in everyday life. Here we will try to give a more detailed picture of these differences. Yet whatever their concrete position on the matter, stakeholders agree that the digital twin must be built with extra consideration for the issue of privacy. We can distinguish the following scenarios in which hazards were linked with the theme of privacy, all of which being amplified by the fact that the digital twin is arguably a *data intense* scenario can multiply the digitalization of information to an unprecedented degree. At one point, as one interviewee put it, the question “Where does *all that data* go?” becomes increasingly important. The following scenarios illustrate the many angles from which the topic of privacy has been approached in our interviews with stakeholders.

In a first risk scenario, patient data is owned by private organisations. In a first scenario, privacy becomes an issue when patient data can be accessed by (or becomes the property of) organisations that might not necessarily put the benefit of the individual values before their own. The prototypical story is that of the insurance company that can make increasingly precise distinctions based on new data points more significant to personal identity than age and pre-existing conditions. The following interviewee impersonated the reaction of an insurance company with access to data from the digital twin.

If we [i.e., the insurance company] know that you’re not that healthy, or that you don’t engage in enough physical activity or you don’t listen to that coach, then we raise your premium, for instance.

In some cases, this first scenario starts as an issue for privacy, but unwound to its final conclusion ends up being a matter of freedom. Breach of privacy is then only the trigger for a more fundamental problem of your own data limiting your own freedom.

A second risk scenario concerns security breaches. The advent of a digital twin can amplify (rather than solve) the hazards of data being lost, leaked or stolen through security breaches or negligence. In a scenario of increased data gathering through digitalization, the depth, and sensitivity of information that “ends up on the street” is much more significant than in the case of the age-old digital patient file. Also, if such a hazard occurs, it is important to know where the responsibility lies. For example, it is difficult to pinpoint at this stage where the digital *is located* – the ownership of servers – an issue that might be important later on when attributing responsibility for security breaches. The presence of genetic information makes these scenarios particularly troublesome, because if the digital twin becomes a more generalized approach, it can become possible to specify, with surprising accuracy, what type of genetic material is better suited for survival and health. Such a scenario can bring back memories of evil intentions concerning political decisions based on convictions about what constitutes a ‘good gene pool’. In light of such discussions, one stakeholder noted, “where it is stored doesn’t really matter because we’ve seen that everything can be hacked!”. And if DNA is accessible through cybercrime, the use of widespread use of DNA profiling in forensic contexts can be exploited through, e.g., placement of DNA samples at crime scenes. If such situations generally constitute a small risk, the widespread need for data in order for the digital twin to function reasonably well considerably amplifies this risk. With this we arrive at what one of our interviewees termed “big data discrimination” where the sheer amount of information that you generate makes it borderline impossible for you to decide where your own privacy norms lie. The patient might end up not being able to “hide”, as the following quote put it:

I wonder. Let’s imagine there are digital twins available. The question is, do I become dependent on a company? And is that an insurance company who can maybe force me to make use of the digital twin or else I don’t get premium reductions? I’m just saying – it *can* happen.

The success of the digital twin is thus potentially their most important enemy, because once they become mainstream, they ‘reach’ new areas of socio-ethical risks. When possibility becomes necessity and when small hazards have catastrophic consequences, the development and spreading of the technology is called into question. As one stakeholder put it, rhetorically: “Are we making ourselves dependent on platforms that we cannot control?” In fact, this situation has already occurred in the case of health insurers imposing a certain speed monitoring technique on vehicles for a lower premium on car insurance – “are we going in that direction?”, asks one of our interviewees.

It should however be mentioned that there are also stakeholders who believe that privacy, while important in general, functions more as a barrier, rather than a starting point, for technological development. To them, privacy appears to function like a golden middle – too little privacy is undesirable because of the previous three scenarios, but too much privacy is equally undesirable because of fields of knowledge that thereby remain untapped. In some cases, the civil society’s urge to increase data protection and to transfer as much as possible the ownership towards the citizen results in a barrier for research and development that is considered by some “unethical [ …] since people might be dying because of this”. In some fields such as cancer research and other diseases with high mortality rates, there is even a question of whether the social, moral and economic benefits of improved treatment do not trump the question of privacy. When asked whether the digital twin will trigger any additional socio-ethical risks compared to current data gathering practices, one stakeholder answered:

I think it’s just standard, standard problems. In principle, once people have cancer, they don’t worry too much about data protection anymore. So data protection is something for healthy people who pretend to be sick. Not for people who are really sick and would like to get fit.

It is important to mention with regard to this fourth scenario that our ethical conceptions of privacy and other individual rights *might change* once the digital twin appears and more so once it becomes mainstream. Some stakeholders see an evolution of the kind we have undergone with the advent of the Internet, such that practices that would have seemed unconceivable (that is, unconceivably *invasive*) twenty years ago are now considered part of our everyday life. What it means to share and thus the limits of reasonable sharing will therefore not stay fixed for the digital twin to incorporate but will rather evolve together with the digital twin. However, whether these intuitive notions will evolve towards a strengthening or weakening of our privacy ensuring regulations is up for discussion.

Finally, the low quality of data is an important risk that can go against the main value quoted in the beginning: patient health, lack of pain, happiness etc. The problem is that the models involved in creating the digital twin, at least at this early stage when artificial intelligence systems must begin their learning from available biomedical data, are themselves fairly fragile. The biomedical sciences are not always seen as being on a par with the truly ‘exact’ sciences even though, of course, there is better and worse research just as in other fields. But in the case of the digital twin, the effects of bad data, bad analysis and, consequently, bad representation, are amplified by the trust we place in those models. One of our respondents sketched the problem as follows:

My biggest fear is that people base the models on academic literature filled with nonsense. [ …] The bigger part of the biomedical sciences is nonsense, there is a lot of bias from researchers, a lot of wrong data. [ …] If people start basing [the digital twin] on this type of data, you’ll never get anything good out of it.

There are various factors that contribute to this decrease in data quality: lack of standardization, publication pressure, bias and the tradition to not publish failed results. The overconfident use of data has resulted in bad decisions in the past without the digital twin and there is no indication that such confidence will diminish once the many spurious variables will be ‘forgotten’ inside a model or project that works with large datasets.

#### Disruption of structures, institutions, and roles

Although the digital twin in health is often presented as an enabling technology - meaning that it comes to merely aid an already existing process as opposed to reshape an entire system (Bagaria et al. 2020; Mesko 2020; Pal 2020) - some of our stakeholders noted risks involved in institutional changes, small as they may appear at first sight. There is, to start with, the question of responsibility of diagnosis once the digital twin intervenes in the diagnostic process. Assuming that the real-life physician remains the diagnostician in charge, what happens if the doctor overrides the (AI-based) component of a digital twin? Equally importantly – what happens when the diagnostic is based on the data from the digital twin and it is the wrong one? Some stakeholders resolve such issues by drastically limiting the ‘reach’ of the digital twin, for example by following the principle that regardless of what the digital twin says, the physician is ultimately the one in charge. The digital twin will present a better picture of different intervention scenarios but the decision to take some route rather than other remains solely on the side of the human physician. Such questions are increasingly brought to the fore if we realize how much more intelligent computers already are and how (organizational, national and international) policy is still mostly lagging behind.

Aside from the question of responsibility, the institution of healthcare is essentially an institution of *contact.* When we are sick, we go to the doctor. The doctor knows *us* to some extent and they exercise that social role partly based on their knowledge of us. The question is thus what the effect is of disrupting such social contacts.

[Digitalization] really affects practices. In the past you had the veterinarian who had a really important role because he interacted closely with the farmers and knew his patients [gives example]. But that role is now excluded because everything is taken care of digitally. And the veterinarian comes around with a bag of pills every now and again, but he is in fact excluded from the process.

At the same time, the contact in question is time-consuming and the growing global population and the increased complexity of diagnostic procedure puts pressure on the system to *reduce* rather than increase the contact in question. This pressure is seen most obviously in the case of the general practitioner who can unload some of the simple cases to the digital twin. But the practice is also foreseen in more complex situations, for example in the case of tumor detection through imaging. Although the digital twin will not “replace the radiologist” but rather “support” her and provide increasingly accurate “insights”, it is at the same time the case that “the radiologist who does not want to work with AI will have a hard time”.

#### Inequality and injustice

There are various ways in which the digital twin can lead to inequality and other forms of injustice. First, as a relatively new technology that is either not accessible to everyone or not covered by health insurance for everyone, the digital twin can act as to widen an already existing socio-economical gap. The simplest thought experiment is that those who have the money to pay for the service of, say, testing a treatment on the digital twin, will be better off because they can access knowledge and expertise that others might not. Second, inequality can result from the implementation of the technology in predominantly North-Western, rich countries with access to the required R&D facilities and the resulting intellectual property. If the technology is not “rolled out” fully to reach Southern and Eastern countries, the digital twin will only increase an already existing global problem of the gap between rich and poor countries. Then the digital twin remains, as one of our interviewees put it, “a story about the few, and not about the many”. Third, inequality can result from the fact that the design of the digital twin will pick up already existing biases since the health system is, as one stakeholder put it, “designed around the white male”. These forms of injustice are already felt in the case of other new and emerging technologies.

Other forms of potential injustice occur when the increased ability to make accurate decisions translates into a decision-making process that becomes increasingly difficult to justify. For example, the mere possibility of determining *with great precision* one’s life expectancy given a certain treatment (based on the digital twin) does not imply that the precision will eventually benefit that person’s health and happiness. Ignorance, in this case, might be bliss. “*Do you indeed* want to know that much about individuals?” In some cases, the stakeholder continue, it will seem unjust to cling to ever smaller differences in, say, life expectancy even though you are capable of measuring them with great accuracy. The digital twin helps us to make accurate claims about a patient, but with nuance comes a certain air of arbitrariness: the fear is that one might fall with some small percentage points on the wrong side of the limit and might end up not qualifying for the surgery or for the insurance coverage. Whereas, lacking accuracy, there was space for negotiation, interpretation and case-by-case judgment.

#### Other socio-ethical risks

Aside from the questions of privacy, disruption and inequality/injustice, several other socio-ethical risks were mentioned. We cannot say whether the interviewees brushed over these issues lightly because they thought these are less important or because they had relatively less knowledge of these aspects (or perhaps both). In any case, the following socio-ethical risks came up as being less pronounced than the previous two:

- Environmental risks stemming from increased transfer and storage of data
- Change in personal identity and responsibility if ‘staying healthy’ is objectified (turned into concrete data points) through Internet of Things application
- The difficulties involved in assessing the reliability and ‘cleanliness’ of data gathered globally or owned by private health companies
- The general danger of imposing the technology under the bargain of ‘better health’, civil disobedience related

### Policy responses

We took it to be self-evident that the goal of stakeholders in this innovation system is that of maximizing the aforementioned socio-ethical benefits while minimizing socio-ethical risks. Asked what would be necessary to create such a ‘best case scenario’, stakeholders seemed to agree on one basic statement: in rolling out the digital twin in health, we must not forget or disregard the values and interests of each of the four helixes (research, policy, civil society and industry). Scenario’s in which one type of value dominate others are typically presented as being of little help in maximizing values and minimizing risks. Collaboration is thus key, yet opinions differed on *how* this collaboration is to be achieved.

We begin with a observed tension between calls to keep the digital twin focused and specialized and calls to broaden its application in order to encourage multi-stakeholder (and thus cross-sectoral) participation. We can refer to these as the KIS (“keep it small”) and the MIB (“make it big”) strategies. The KIS strategy is backed by reference to the great number of uncertainties that the digital twin still holds. In light of these uncertainties, it is better to limit the digital twin idea to already existing fields, or to slowly absorb new related fields. For proponents of the KIS strategy, the idea of a total digital twin is a chimera that should not be pursued to seriously for fear of both practical and socio-ethical risks.

Digitalization, data sharing, privacy protection, good algorithms, validations, making sure that nobody is excluded, not forcing it – if you keep it small, directed at certain problems, it can work.

On the other side, the MIB strategy asks for a broad collaboration between various fields of medicine as well as various sectors (or ‘helixes’) precisely in order to achieve the type of collaboration that would responsibly include multiple types of applications, value systems and perspectives. Supporters of this strategy call for “big consortia” and for a corroboration of data that is now produced specifically in one field of medicine. According to supporters of this strategy, various fields of medicine are constantly generating all sorts of data and “they’re not doing anything with it” whereas if you put all that together you can eventually create a digital twin that is more comprehensive than, say, only data from imaging. The goal, according to the MIB strategy, is to be able to have a dataset *for the individual* as a whole, not for specific organs or functions. In some cases, the smallness of the digital twin industry appears to be somewhat surprising:

We just need more funding if we want to do the same type of technological breakthroughs which we aim for in other areas. We have to put more money behind it. My country is paying probably 100 billion of the tax payers’ money to make solar cells cheaper and more effective. [ …] If we were prepared to do something similar with cancer patients, we would have the same effect, an enormous acceleration. Things would get very rapidly much better and much cheaper. But for some reason it’s the same arguments which people find extremely convincing for solar cells, don’t seem to work as well when you’re a survivor to cancer.

To be sure, numerous legal and economic hurdles still need to be addressed in this development. On the legal side, policy is primarily perceived as lagging behind. In most cases, this means that policy is written *post facto* after hazards take place (for example, policy regarding data protection did not coincide with the advent of the Internet in the late 2000s). Some respondents speak of an “enormous lag” between the R&D developments and policy. One of our respondents mentioned an interesting case where stakeholders involved in R&D explicitly *requested* regulation from and international legislative bodies since they recognized the social and economic benefits of a clear framework for action.

It was not just scientists but also industry players who went together to the European Commission and asked: *Regulate us!* Which never happens normally, right? Because in general you think: the less legislation there is, the more freedom you have. But in this case everyone saw the benefits of regulation. [ …] We protect both the patients and ourselves. It’s in the benefit of the entire field.

By contrast, some respondents perceive the environment as being populated by too many regulations. Most countries “live in the analogue world” and this impacts their policy-making abilities when it comes to technologies that use, e.g., artificial intelligence:

Most of the organisations that approve and support new research and development are still in the analogue view of the world. In a digital environment, collecting data and analyzing data is much faster, the outcome can be created faster. But then there is always this question: how did you come to the outcome, can you explain to us [regulators] how you created this outcome and is it a reproducible set of steps? [ …] A new view of how you establish digital technologies is something that is missing.

On the economic side, the hurdle consists in the policymakers and investors willingness to put their trust in a development that is still uncertain. The healthcare system is described as being “hugely underfunded”. In some cases, the digital twin, working with the very big or the very exact, might suffer from an image problem. Stakeholders outside the R&D process might be inclined to dismiss cutting-edge technologies as something that is either extremely costly (disregarding the cost-saving effects of improved treatment) or extremely improbable (thinking that the digital twin is all but science fiction).

But I think that the point is that a lot of what we are working on sounds for them much too much as science fiction.

Various other barriers have been mentioned and discussed during our workshop with experts that took place in December 2020. For example, there is first an epistemic challenge. We are particularly good at describing processes within industry, but significantly less equipped to characterize molecular processes. Part of this epistemic deficit has to do with the fact that the human processes that we would like to model cannot be measured in the lab so they need to be reverse engineered through what is essentially a trial and error approach. The epistemic problem should not be underestimated. As one respondent put it, rather pessimistically, if we notice that it took a long time and to arrive at a fair understanding of airplanes engines (which are, in essence, fairly simple rotary machines), we should expect the digital twin to be exponentially more difficult because any human organ is indefinitely more complex than the most complex airplane engine. This adds an essential note of realism to the current enthusiastic messages about the advent of the digital twin. Finally, we should also mention a lack of clarity and focus. In the case of digital twins, the starting point is clear (digitalization and algorithms), the endpoint is also clear (better healthcare, less disease), but the sheer number of possibilities to arrive from one to the other at this point constitutes a barrier. The digital twin appears at times as a solution to just about anything (see the list of applications in the introduction). While this might work in favor of developing new research areas and funding R&D, the dough might sometimes be stretched too thin. An embarrassment of possibilities for the use of the digital twin is not necessarily a fruitful setting for specific breakthroughs, even though it is a rich context with enough space for everyone. Similar problems have been encountered in the area of the human genome, where a lack of concrete goal and parameters for success (a concrete disease, a concrete field of application) slowed down the R&D process.

## Discussion

The digital twin has a marvelously ambivalent status. It is at the same time a buzzword everyone is eager to use and a niche preoccupation of which only a happy few can claim ownership. It is at the same time an umbrella term and an element of a highly technical lingo. The fact that the digital twin is a technological cocktail, containing uncertain combinations of already existing technologies, tends to increase this ambivalence. We nevertheless endeavored to study the socio-ethical contours of this technology *now* seeing that much literature of public engagement and responsible innovation stresses the importance of “upstream” intervention (Pidgeon and Rogers-Hayden 2007; Wilsdon and Willis 2004). The risk of talking at cross purposes between individuals that simply use the term ‘digital twin’ differently was partly mitigated by the use of the same definition both in the interviews and in our workshop discussions. However, as pointed out in the previous section, the question of what the expression ‘digital twin’ means was more often than not the first point of discussion with our respondents. This fluctuation of meaning is not uncommon in the case of emerging technologies (see Sandler 2014), but it must for that reason not be forgotten when generalizing based on results or creating technology-specific policy.

By asking respondents to rank their own socio-ethical themes, it is clear that we have managed to distill the more urgent themes that the digital twin will bring in healthcare in the upcoming decade. At the same time, it is difficult to investigate the basis on which the respondents made their decisions. Rating urgency (or ‘importance’) of a socio-ethical theme might vary among respondents. For example, a stakeholder might consider theme A to be urgent because nobody is addressing it (urgency by neglect) while another stakeholder might consider theme B to be urgent because everybody is addressing it (urgency by attention). It was only in coding the interviews that we realized that interviewees can differ in this regard. To formulate the point more generally, terms such as ‘importance’ and ‘urgency’ have a difficult relationship with the public agenda. The urgency of a point can be deduced by its high placement on the public agenda but it can also be deduced from its low placement on the agenda. It is not immediately clear to us how the interviewees navigated this ambiguity, nor is it apparent from their justification of answers which routes they took.

A second point concerns the distribution of respondents per helix. Our sample was clearly dominated by the research-industry combination, together making up almost 70% of our respondents. According to the quadruple helix theory, these two helixes are typically directed towards the creation of academic value (answers to research questions and new questions to be answered) and business value (products to be sold). It is therefore plausible that the benefits and risks highlighted in this study are primarily the ones that are more visible or more urgent *as seen primarily from these two helixes.* It should be noted, however, that the types of socio-ethical risks brought to the fore by these respondents are not the ones that constitute dangers exclusively for the production of academic and business value. Privacy issues, social disruption, security – these are not exclusive threats in the academic and business world. We could say that, at least prima facie, the respondents considered the impact of the technology on the health innovation system as a whole, including all the four helixes. Thus, although the quadruple helix theory was efficient as a selection tool, we should not assume that the selected individuals were necessarily performing only as representatives of their ‘helix of origin’. A full analysis of problems *per* helix will have to include questions that ascribe to participants the task of discussing socio-ethical themes specifically from the perspective of their helix and then comparing the results. Such an analysis was not undertaken in the present study.

Several remaining point regarding the theoretical apparatus. Brey’s categories, despite their great heuristic value (meaning that they are of help to organize or trigger respondents’ thought), still behave as a rough-and-ready list that shows little guiding principles. Some of the problems concerning consistency have already been mentioned in Socio-ethical scan for the emerging digital twin section, but even the abridged version we ended up using is not without its problems. We say this in light of the many discussions that we had with interviewees, urging them to ignore possible overlaps and unclarities, but also in light of commonsense objections. For example, how can ‘health’ be a category alongside ‘pain and suffering’ if lack of health typically results in some form of pain and suffering? The term ‘happiness’ could easily include everything else (at least in an Aristotelian reading of the list). Also, some of the terms included are hopelessly vague such that even a very charitable reading of someone who intends to use the list heuristically will likely cause some irking. What social institutions are supposed to be ‘vital’, what goods are supposed to be ‘primary’? What is animal welfare and why is it separated from animal rights? It is not particularly clear where the answers must come from in an empirical study. Should the interviewer impose her meaning (risking bias) or should everyone be allowed to supply their own meanings (risking incomparability between data points)?

In light of our use of the list, it seems to us more expedient to supplement the list with a series of prototypical scenarios that are technology and field-independent. These scenarios are, we suspect, closer to how subjects approach those terms and are thus likely to avoid at least some of the definitional issues. In order to maintain the heuristic function of the list, these scenarios can be formulated as challenges. We give several examples in Table 3.

**Table 3:** Scenarios accompanying the ‘Brey categories’

Topics	Challenge
• Impact on individuals ○ Health and body ○ Pain and suffering ○ Psychological effect … .	- Name an individual or group of individuals who might suffer more (or less) because of the technology. - Explain their increased (or diminished) suffering - Name one concrete thing that we couldn’t do before but we can do now - Name one way in which the environment will benefit (or be damaged) by this technology
• Rights ○ Freedom ○ Autonomy ○ Human dignity ○ Privacy ○ Property …	- Look at the list of human rights in the left column. Freedom refers to a situation where you should be able to do *x* but some authority will not allow it. Can you describe an actual or imaginary situation where freedom is violated? Can you describe an actual or imaginary situation where freedom is enhanced? Etc.

Turning to the quadruple helix theory, as important as we found the four-fold distinction for recruiting respondents, we ended up doubting almost every respondent’s belonging to one category or the other. Although we eventually assigned each respondent to a certain category, doubts could have been placed on virtually every respondent. The problem seems to be the following. The four helixes are defined based on individuals’ background or sector of origin. If an individual works at an organisation that, say, makes profit and is listed on the stock exchange, then the theory seems to suggest that the person in question represents industry. But there are two unwarranted assumptions there. First, the assumption that the individual’s work is directed solely (or primarily) at making profit and that the individual’s internal and external rewards are based on it. But an industry organisation that directs its funds towards solving societal problems is at the same time (by definition) a member of civil society or at least acting like one. Similarly, if the company makes its profit through research and development then it should also cover a part of the research helix. In short, as soon as we start looking at the *activity* of the individual in question, any ‘clean-cut’ assignment to one category or the other becomes impossible. It seems to us more expedient to think of the four helixes not so much as sectors where individual belong because of their identity, but rather as collections of activities that are directed towards specific types of value. In this way, our imaginary individual mentioned above might ‘cover’ all four helixes at the same time, while doing so more for some helixes than the others.

**Fig. 1:**
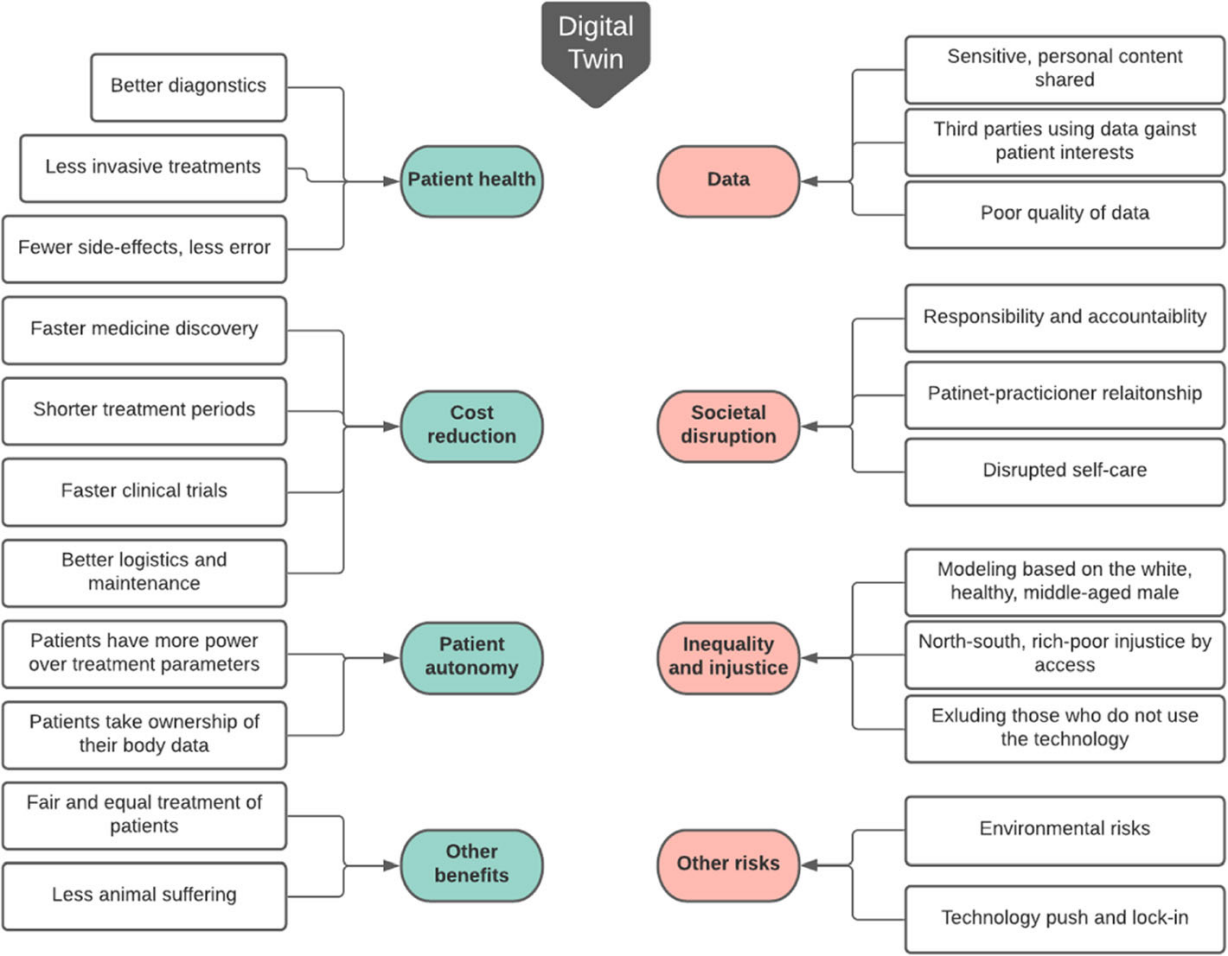
Overview of identified socio-ethical issues

In Fig. 1 we provide an overview of the discussed results.

## Conclusion

Let us go back to the philosophical doppelgangers that opened the present study. What distinguishes them from digital twins is the sheer level of accuracy and detail. For example, when Donald Davidson is hit by a lightning bolt an exact replica of Donald Davidson is marvelously created after disintegrating the original one. This replica is in all respects identical to the original save of course for the way it came into being. The real digital twin is less accurate but, in exchange, more realistic regarding its birth certificate. The real digital twin is born by combining existing and emerging technologies such as AI, Internet of Things, big data and robotics.

The present study has shown that the digital twin is still, at the current technology level, almost indistinguishable from already-existing efforts to digitalize the healthcare process. The digital twin might of course *in theory* different from the (less emerging, more established) digital model or digital shadow. But until the connection between the patient and the model is brought to life, the two worlds will share socio-ethical benefits and socio-ethical risks. Content, quality and ownership of data is central to these efforts and given the importance stakeholders attach to these issues, it might not be farfetched to say that if these issues are solved, the digital twin will penetrate the socio-ethical system fast and with the support of all helixes. Other socio-ethical risks that can hamper these developments do not seem to be deal-breakers. For example, the technology can exacerbate an already-existing distance between those with access to, and those far removed from, latest types of healthcare, but such issues did not appear, in our interviews at least, to constitute major road blocks.

## Data Availability

Data cannot be made available in public repositories due to Informed Consent agreement. The datasets during and/or analysed during the current study available from the corresponding author on reasonable request.

## Abbreviations

ATE: Anticipatory Technology Ethics
IoT: Internet of Things
R&D: Research and Development

## Footnotes

1 We see, then, that the *automata* of Descartes is conceptually different from the digital twin since it does not allow for a constant relationship between the robot and its counterpart.
